# Life in lockdown: a longitudinal study investigating the impact of the UK COVID-19 lockdown measures on lifestyle behaviours and mental health

**DOI:** 10.1186/s12889-022-13888-1

**Published:** 2022-08-05

**Authors:** Emma Solomon-Moore, Jeffrey Lambert, Elisabeth Grey, Fiona Gillison, Nick Townsend, Betty Busam, Kyriakos Velemis, Christopher Millen, Fran Baber, Tania Griffin

**Affiliations:** 1 Department for Health, University of Bath, Claverton Down, Bath, BA2 7AY UK; 2 Bristol Medical School (Population Health Sciences), University of Bristol, Bristol, UK; 3 Department of Psychology, University of Bath, Claverton Down, Bath, BA2 7AY UK; 4 Department of Psychology, University of Heidelberg, Grabengasse 1, 69117 Heidelberg, Germany

**Keywords:** COVID-19, Coronavirus pandemic, Lockdown restrictions, Lifestyle behaviours, Physical activity, Diet, Mental health, Longitudinal

## Abstract

**Background:**

The COVID-19 pandemic led to the UK government enforcing lockdown restrictions to control virus transmission. Such restrictions present opportunities and barriers for physical activity and healthy eating. Emerging research suggests that in the early stages of the pandemic, physical activity levels decreased, consumption of unhealthy foods increased, while levels of mental distress increased. Our aims were to understand patterns of diet, physical activity, and mental health during the first lockdown, how these had changed twelve-months later, and the factors associated with change.

**Methods:**

An online survey was conducted with UK adults (*N* = 636; 78% female) during the first national lockdown (May–June 2020). The survey collected information on demographics, physical activity, diet, mental health, and how participants perceived lifestyle behaviours had changed from before the pandemic. Participants who provided contact details were invited to complete a twelve-month follow-up survey (May–June 2021), 160 adults completed the survey at both time-points. Descriptive statistics, T-tests and McNemar Chi Square statistics were used to assess patterns of diet, physical activity, and mental health at baseline and change in behaviours between baseline and follow-up. Linear regression models were conducted to explore prospective associations between demographic and psycho-social variables at baseline with change in healthy eating habit, anxiety, and wellbeing respectively.

**Results:**

Between baseline and follow-up, healthy eating habit strength, and the importance of and confidence in eating healthily reduced. Self-rated health (positively) and confidence in eating healthily (negatively) were associated with change in healthy eating habit. There were no differences between baseline and follow-up for depression or physical activity. Mean anxiety score reduced, and wellbeing increased, from baseline to follow-up. Living with children aged 12–17 (compared to living alone) was associated with an increase in anxiety, while perceiving mental health to have worsened during the first lockdown (compared to staying the same) was associated with reduced anxiety and an increase in mental wellbeing.

**Conclusions:**

While healthy eating habits worsened in the 12 months since the onset of the pandemic, anxiety and mental wellbeing improved. However, anxiety may have increased for parents of secondary school aged children.

**Supplementary Information:**

The online version contains supplementary material available at 10.1186/s12889-022-13888-1.

## Background

The COVID-19 pandemic has led to unprecedented measures worldwide to control virus transmission. In the UK, on March 23rd 2020, the government announced a nationwide lockdown ordering the public to stay home and leave only for a limited number of reasons, including for exercise (once a day only), to purchase household essentials, for a medical emergency, or to go to work if classed as a key worker (e.g., emergency services, healthcare workers, food delivery drivers). All non-essential businesses were closed and visiting family or friends outside the individual’s household was prohibited. Over time, the initial lockdown was eased in stages with home-nation variations; including being allowed to leave the house to exercise more than once a day, the opening of non-essential shops, and the opening of the hospitality sector. Additional regional and national lockdowns and restrictions were enforced throughout Autumn-Winter 2020–2021, with lockdown restrictions gradually eased across Spring 2021, and almost all restrictions removed in July 2021.

Previous research has highlighted the complex associations between diet, physical activity, and mental health and wellbeing [1–6], and the influence that environmental cues can have on lifestyle choices [7–10]. It is well established that physical activity and good nutrition have important physical and mental health benefits [1, 2, 5, 11–13]. The COVID-19 lockdown restrictions have presented opportunities as well as barriers for physical activity and healthy eating habits, and research from around the world is emerging on the impact of COVID-19 control measures on lifestyle behaviours and mental health [14–22].

Several studies have observed a reduction in physical activity levels through the start of the pandemic [14–16]. A large study using daily step count measurements from smartphone accelerometers provided by 455,404 users from 187 countries within 30 days of the pandemic being declared, identified a 27.3% decrease in mean steps worldwide [16]. Regional variation was evident, for example, in Italy - which declared a nationwide lockdown - a 48.7% maximal decrease in steps was found, whereas in Sweden, where social distancing and limitations on gatherings were advocated rather than legally enforced, there was a 6.9% maximal decrease. Even in countries that did not institute lockdowns people still exhibited decreases in overall step count, suggesting that social distancing measures or concerns for health related to the pandemic, may have had a negative effect on overall physical activity [16]. A cross-sectional survey of Italian adults (*n* = 2524) suggested that self-reported physical activity decreased in all age groups during the first phase of the COVID-19 pandemic (Mean: 2429 vs. 1577 metabolic equivalent task minutes per week, *p* < 0.0001) [15]. However, the study was limited by its reliance on participant recall of physical activity behaviour from before the COVID-19 pandemic. Overall, the emerging research signalled that in the early stages of the pandemic physical activity levels decreased.

Looking at physical activity alongside other lifestyle factors including diet, an international cross-sectional survey examined lifestyle changes that occurred during COVID-19 lockdowns in 1047 adults primarily from Western Asia, North Africa and Europe [14]. This study found self-reported levels of physical activity and alcohol binge drinking decreased, while sedentary time, consumption of unhealthy food, eating out of control, and snacking between meals increased during the lockdowns [14]. In an observational retrospective study, Pellegrini and colleagues [17], examined changes in weight and nutritional habits in 150 Italian adults with obesity during the COVID-19 lockdown period. Mean self-reported weight gain was 1.5 kg, with lower education levels, self-reported anxiety/depression, and not consuming healthy foods positively associated with weight gain [17]. Another study examined dietary changes during the COVID-19 lockdown in Spain by examining food purchases, finding that energy intake increased by 6% while nutritional quality decreased by 5% compared to pre-COVID-19 eating patterns [18]. At the time of writing, however, few published studies have focused on physical activity, diet and mental health in combination.

The pandemic and control measures have had an impact on people’s mental wellbeing. In a secondary analysis of the UK Household Longitudinal Study (UKHLS) panel (*n* = 42,330), population prevalence of clinically significant levels of mental distress in adults rose from 18.9% in 2018–19 to 27.3% in April 2020, 1 month into UK lockdown [19]. Increases in mental distress were also found to be greatest for those aged between 18 and 34 years old, women, and people living with young children [19]. In an online survey of 1005 Austrian adults, depressive symptoms (21%) and anxiety symptoms (19%) were higher during the COVID-19 lockdown compared to a large Austrian survey conducted before COVID-19 [20]. Similarly, in a survey of 1210 adults in China [21], 53.8% rated the psychological impact of the outbreak as moderate or severe, 16.5% reported moderate to severe depressive symptoms, and 28.8% reported moderate to severe anxiety symptoms. While there is some research available on how COVID-19 lockdown restrictions have had an impact on mental health for UK adults [19, 22], data are limited, and not enough is known about potential long-term effects of the pandemic.

The emerging evidence highlights the impact of the varied COVID-19 restrictions on lifestyle behaviours and mental health across the globe. However, much of the research to date has relied on cross-sectional data in the immediate aftermath of the pandemic, thus, it would be useful to explore how diet, physical activity and mental health have changed throughout the course of the pandemic in order to understand and respond to the likely long-term impact on health and wellbeing. Therefore, the current study aimed to use longitudinal survey data to explore the following research questions a) what were the patterns of lifestyle behaviour in the UK during the initial COVID-19 lockdown measures?, b) how diet, physical activity, and mental health changed between the first UK lockdown measures and twelve-months later?, and c) what factors were associated with change in diet, physical activity and mental health between baseline and twelve-month follow-up?

## Methods

All methods were carried out in accordance with relevant guidelines and regulations. An online survey focusing on physical activity, diet and mental health was hosted using JISC Online Surveys (see supplementary materials). The survey was promoted through social media (Twitter and Facebook), a press release and interviews with local radio stations. The survey was open to all adults aged 18 years and over living in the UK through the COVID-19 lockdown measures as long as they could read, write and understand written English and had capacity to provide informed consent to participate. Upon accessing the survey link, participants were asked to read the information sheet and complete an online consent form to access the survey. The survey was open during the first national lockdown from May 7th to June 14th 2020, with the closing date reflecting a change in lockdown restrictions with non-essential shops opening on June 15th 2020.

Participants were able to choose to complete the survey anonymously or, if they were interested in completing any additional elements of the study (follow-up survey 12 months from baseline ora semi-structured qualitative interview), they could provide their contact details at the end of the baseline survey. Participants who provided their contact details were emailed with a link to the diet recall and an invitation to contact the team if they were interested in taking part in an interview. The methods and results from the qualitative interview study are presented elsewhere. The study received ethical approval from the University of Bath Research Ethics Approval Committee for Health (REACH).

A follow-up survey was scheduled to take place 12 months after the initial survey. On January 6th 2021, with COVID-19 cases rising, England entered its third national lockdown. The government set out a roadmap to gradually ease restrictions, including groups of six being able to meet outdoors (March 29th 2021), non-essential retail and outdoor hospitality reopening (April 12th 2021), increased social contact indoors and outdoors and indoor hospitality reopening (May 17th 2021), and a planned removal of all social contact restrictions (June 21st 2021), although this was delayed (July 19th 2021). Data were collected for the twelve-month follow-up survey between May 23rd and June 20th 2021, where indoor socialising was permitted but some restrictions were still in place. Participants who provided their contact details when completing the baseline survey were emailed a link to the follow-up survey. Participants were provided with an anonymised ID number that they were instructed to enter when completing the follow-up survey so that their data could be matched with their baseline survey data.

### Baseline survey measures

The baseline survey was used to collect demographic information and self-reported physical activity, diet, and mental health during the first UK lockdown, as well as how participants perceived these lifestyle behaviours had changed from before the pandemic.

### Demographic measures

Demographic questions included gender, age category, ethnic group, and number/relationship of other people living in the household. Participants provided their postcode to determine which part of the UK they resided in, and this was also used to assign Indices of Multiple Deprivation (IMD) scores, based upon the English Indices of Deprivation (http://data.gov.uk/dataset/index-of-multiple-deprivation). Participants were asked to report: their general health on a five-point scale (from excellent to poor); whether they are classed as high risk for COVID-19; and their working situation during the initial COVID-19 lockdown measures (i.e., not working, working from home, working outside of home but socially distanced, or a frontline NHS or key worker not able to socially distance).

### Physical activity measures

Physical activity behaviour was self-reported using the nine-item International Physical Activity Questionnaire - Short Form (IPAQ-SF) [23]; participants reported the time they spent engaging in walking, moderate-intensity, and vigorous-intensity physical activity across the last 7 days. The amount of time participants spent walking (at a brisk or fast pace) and engaging in moderate-to-vigorous-intensity physical activity per week was used to determine whether participants met current UK physical activity guidelines (i.e., 150 minutes per week of moderate-to-vigorous-intensity physical activity) [24]. Participants were asked to report whether their physical activity had changed during the initial lockdown, and if so, whether it had ‘increased’, ‘decreased’, or ‘neither increased nor decreased, but was just different’. Additionally, participants were asked to rate how important they thought it was to be physically active during the lockdown period, on a scale from 1 ‘not at all important’ to 10 ‘very important’, as well as how confident they were that they could be physically active during the lockdown period from 1 ‘not at all confident’ to 10 ‘very confident’. These items were based on measures in the International Health and Behaviour Survey (adapted from [25]).

### Diet measures

Participants were asked whether their diet had changed during the initial lockdown, and if so, whether it had ‘improved during lockdown’, ‘worsened during lockdown’, or ‘neither improved nor worsened, just different’. The survey included a measure to assess participants’ habit strength for healthy eating using the 4-item Self-Report Behavioural Automaticity Index (SRBAI) [26], adapted for healthy eating. The SRBAI asked participants to rate their agreement to four statements (e.g., Deciding to eat healthy foods is something I do automatically) on a seven-point scale from 1 ‘completely disagree’ to 7 ‘completely agree’. Scores for the individual items were averaged to create a mean healthy eating habit score (potential range 1–7), with higher scores representing a stronger healthy eating habit. Participants were also asked to rate how important they thought it was to eat a healthy diet during the initial lockdown period, on a scale from 1 ‘not at all important’ to 10 ‘very important’, as well as how confident they were that they could eat a healthy diet during the lockdown period from 1 ‘not at all confident’ to 10 ‘very confident’ [25].

### Mental health measures

To measure prevalence of current depression symptoms, the validated eight-item Patient Health Questionnaire depression scale (PHQ-8) was used [27]. The PHQ-8 measures depressive symptoms (e.g., little interest or pleasure in doing things) across the last 2 weeks on a four-point scale from 0 ‘not at all’ to 3 ‘nearly every day’. The PHQ-8 has a total score range from 0 to 24, where scores of 5, 10, 15, and 20 represent cut-points for mild, moderate, moderately severe and severe depression. Participants were dichotomised into: < 10 ‘none to mild depression’ and > =10 ‘moderate to severe depression’. The PHQ-8 has shown good reliability and validity [27]. To measure current anxiety levels, the validated General Anxiety Disorder-7 scale (GAD-7) was used [28]. Participants responded to seven items on their anxiety symptoms (e.g., feeling nervous, anxious or on edge) across the last 2 weeks on a four-point scale from 0 ‘not at all’ to 3 ‘nearly every day’. Total score range for the GAD-7 is 0–21, with scores of 5, 10, and 15 taken as cut-points for mild, moderate and severe anxiety. Participants were dichotomised into two categories: < 10 ‘minimal to mild anxiety’ and > =10 ‘moderate to severe anxiety’. The GAD-7 has shown good reliability and validity [28]. Wellbeing was measured using the Short Warwick-Edinburgh Mental Wellbeing Scale (SWEMWBS,©NHS Health Scotland, University of Warwick and University of Edinburgh, 2008, all rights reserved). SWEMWBS asks participants to respond to seven statements (e.g., I’ve been feeling optimistic about the future) to describe their experience over the last 2 weeks on a five-point scale from 1 ‘none of the time’ to 5 ‘all of the time’. SWEMWBS scores are summed with total scores ranging from 7 to 35, with higher scores indicating higher positive mental wellbeing. Participant scores were dichotomised into two groups: > = 28 for ‘high mental wellbeing’ and < 28 for ‘low to moderate mental wellbeing’. Participants were also asked to report whether their mental health had changed during the initial lockdown, and if so, whether it had ‘worsened’, ‘improved’ or ‘neither improved nor worsened, just different’. The SWEMWBS has shown good performance as an instrument to measure wellbeing with good reliability and validity [29].

### Twelve-month follow-up survey measures

The measures included in the follow-up survey closely matched the baseline survey. Participants were asked to rate their general health, and whether anything had changed regarding their household or working situation since the baseline survey. In terms of physical activity, participants were asked to report their physical activity behaviour using the IPAQ-SF [23], how important they felt it was to be physically active over the coming month, and how confident they were that they could be physically active over the coming month [25]. In relation to diet, participants were asked to report their habit strength for healthy eating (SRBAI) [26], how important they thought it was to eat a healthy diet over the coming month, and their confidence in eating a healthy diet over the coming month [25]. In terms of their mental health, participants were asked to repeat the PHQ-8 [27], GAD-7 [28], and SWEMWBS (©NHS Health Scotland) scales.

### Data analysis

Descriptive statistics (means, standard deviations, proportions) were used to examine the distributions of demographic, diet, physical activity, and mental health variables for the baseline survey sample. For the participants who completed the survey at both time-points, paired sample T-tests (continuous variables) and McNemar Chi-Square tests (categorical variables) were conducted to test whether demographic, diet, physical activity, and mental and physical health variables differed between baseline and twelve-month follow-up.

Univariate and multivariate linear regression models were used to calculate prospective associations between predictor variables (i.e., demographic and psycho-social) at baseline with change in outcome variables (i.e., lifestyle behaviours and mental health) between baseline and twelve-month follow-up. Outcome variables were as follows: change in healthy eating habit (as a proxy for dietary behaviour [26]), change in physical activity (minutes per week of moderate-to-vigorous physical activity), change in depression (PHQ-8 summary score), change in anxiety (GAD-7 summary score), and change in mental wellbeing (SWEMWBS summary score). Change scores for the outcome variables were calculated by subtracting the baseline score from the twelve-month follow-up score, where a negative score would indicate a reduction in the outcome of interest. Univariate analyses were used to model the effect of each predictor variable on each of the outcome variables. Any significant associations in the univariate models were then entered into multivariate models for each of the outcome variables. If t-test statistics for the difference between baseline and twelve-month follow-up for any of the outcome variables were non-significant (*p* < 0.05), prospective linear regression analyses were not conducted. All analyses were conducted in STATA version 16 (StataCorp, 2019).

## Results

### Baseline sample characteristics

At baseline, 636 eligible participants completed the online questionnaire. Compared to the general UK population (50.6% female [30]; 8.4% living in South-West England [31]), most participants were female (78.0%) and from South-West England (75.3%), with the remaining participants from South-East England (12.1%), West Midlands (2.3%), East Midlands (1.9%), Scotland (1.9%), Wales (1.8%), and other regions of the UK (4.7%). Overall, 91.7% of the sample identified as White British, 5% identified as being from other White backgrounds, with the remaining 3.3% from other ethnic backgrounds. Census data from 2019 revealed that 78.4% of the population of England and Wales identified as White British [32]. Compared to the UK adult population (27.8, 48.9 and 23.6% respectively) [30], 38.1% of the sample were aged 18–34 years, 51.7% aged 35–64 years, and 10.2% aged 65 years and over. Table 1 shows the characteristics of the adults who participated in the online survey at baseline.

**Table 1:** Characteristics of participants who completed the baseline survey during the first COVID-19 lockdown (*N* = 636)

	N	Mean (SD) or %
**Demographic characteristics**		
Gender – % female	631	78.0%
Age category (%)	636	
*18–34 years*		38.1%
*35–64 years*		51.7%
*65+ years*		10.2%
Index of Multiple Deprivation quintile (%)	521	
*1–2 (less deprived)*		58.2%
*3, 4 & 5 (more deprived)*		41.8%
Self-rated health during first COVID-19 lockdown (%)	633	
*Poor / Fair*		13.9%
*Good*		29.9%
*Very good / Excellent*		56.2%
Proportion classed as high risk for COVID-19 (%)	633	14.7%
Living situation during first COVID-19 lockdown (%)	634	
*Living alone*		12.2%
*Living with others (not dependents)*		43.1%
*Living with children aged 12–17*		9.3%
*Living with children aged 0–11*		20.5%
*Living with someone at risk of COVID-19*		15.0%
Working situation during first COVID-19 lockdown (%)	636	
*Not working*		37.3%
*Working from home*		35.1%
*Working outside home, but socially distanced*		7.7%
*Frontline NHS workers or key workers*		15.6%
**Diet variables**		
Healthy eating habit score (1–7)	593	4.59 (1.65)
Importance of eating healthily during lockdown (1–10)	634	8.89 (1.52)
Confidence in eating healthily during lockdown (1–10)	636	7.60 (2.17)
Perceived change in diet during first COVID-19 lockdown (%)	636	
*Diet worsened*		23.1%
*Diet stayed the same*		57.9%
*Diet improved*		19.0%
**Physical activity variables**		
Importance of being physically active during lockdown (1–10)	636	9.02 (1.40)
Confidence in being physically active during lockdown (1–10)	633	7.10 (2.61)
Moderate-to-vigorous physical activity (minutes per week)	625	424.39 (420.67)
Proportion meeting physical activity guidelines (≥150 minutes/week)	625	67.7%
Perceived change in physical activity during first COVID-19 lockdown (%)	633	
*Physical activity decreased*		33.6%
*Physical activity stayed the same*		32.2%
*Physical activity increased*		34.1%
**Mental health variables**		
Depression PHQ-8 score (0–24)	621	6.72 (5.36)
Proportion with moderate-to-severe levels of depression (score ≥ 10, %)	621	25.3%
Anxiety GAD-7 score (0–21)	625	5.68 (5.08)
Proportion with moderate-to-severe levels of anxiety (score ≥ 10, %)	625	20.5%
Mental wellbeing SWEMWBS score (7–35)	628	22.71 (4.70)
Proportion with high levels of mental wellbeing (score ≥ 28, %)	628	16.2%
Perceived change in mental health during first COVID-19 lockdown (%)	634	
*Mental health worsened*		33.8%
*Mental health stayed the same*		58.7%
*Mental health improved*		7.6%

At baseline, participants reported moderate-to-strong healthy eating habits (Mean = 4.59, SD = 1.65), placed high importance on eating healthily during lockdown (Mean = 8.89, SD = 1.52), and moderate confidence in their ability to do so (Mean = 7.60, SD = 2.17; Table 1). Twelve months later, 23.1% perceived their diet had worsened, 57.9% perceived their diet had stayed the same, while 19.0% perceived their diet had improved.

Similar to diet, participants felt it was very important to be physically active during lockdown (Mean = 9.02, SD = 1.40), but had moderate levels of confidence for doing so (Mean = 7.10, SD = 2.61; Table 1). On average, participants reported engaging in high levels of moderate-to-vigorous-intensity physical activity, but there was wide variation between participants (Mean = 424.39, SD = 420.67). This equated to two-thirds of participants engaging in sufficient physical activity to meet the UK government’s recommended guidelines [24]. The sample were roughly evenly split on whether they perceived their physical activity had decreased (33.6%), stayed the same (32.2%), or increased (34.1%) since the lockdown started.

One quarter of participants (25.3%) reported moderate-to-severe levels of depression, one fifth (20.5%) reported moderate-to-severe levels of anxiety, while 16.2% reported high levels of mental wellbeing (Table 1). Only 7.6% of participants perceived that their mental health had improved since lockdown restrictions started, while 58.7% perceived their mental health had remained the same, and 33.8% perceived their mental health had worsened (Table 1).

### Longitudinal changes in diet, physical activity and mental health variables

At twelve-months follow-up, 414 participants who provided their contact details at baseline were emailed the link to the follow-up survey, of whom 160 completed the follow-up survey (response rate: 38.6%). The twelve-month follow-up sample were generally representative of the baseline sample but tended to be older (20.0% compared to 10% were aged 65+ years), were less likely to live in a deprived area (63.8% compared to 58.2% in less deprived quintiles 1 & 2) and were more likely to be classed as a high risk for COVID-19 (20.9% responded yes).

### Diet

Healthy eating habit score (T = 4.53, *p* = < 0.001, d = 0.33), importance of eating healthily (T = 2.19, *p* = 0.029, 0.17), and confidence in eating healthily (T = 2.76, *p* = 0.006, d = 0.25) showed small reductions between baseline and twelve-month follow-up (Table 2).

**Table 2:** Differences in lifestyle behaviour variables for participants who completed the survey at both time-points (*N* = 160)

	Baseline		12-month follow-up		T-test/McNemar^a^
	N	Mean (SD) or %	N	Mean (SD) or %	T or X^**2**^ (***p***)
**Diet variables**					
Healthy eating habit score (1–7)	132	4.95 (1.66)	132	4.40 (1.69)	4.53 (< 0.001)
Importance of eating healthily during lockdown/ over the coming month (1–10)	158	9.09 (1.46)	158	8.78 (1.78)	2.19 (0.029)
Confidence in eating healthily during lockdown/ over the coming month (1–10)	159	7.92 (2.17)	159	7.37 (2.17)	2.76 (0.006)
**Physical activity variables**					
Importance of being active during lockdown/ over the coming month (1–10)	158	9.20 (1.33)	158	9.07 (1.43)	1.20 (0.230)
Confidence in being active during lockdown/ over the coming month (1–10)	157	7.07 (2.68)	157	7.09 (2.79)	−0.08 (0.939)
Moderate-to-vigorous physical activity (minutes per week)	152	434.14 (400.11)	152	438.71 (502.22)	−0.11 (0.912)
Met physical activity guidelines (≥150 minutes/week, %)	152	69.7%	152	65.8%	0.75 (0.387)
**Mental and physical health variables**					
Depression PHQ-8 score (0–24)	145	5.70 (4.90)	145	5.09 (5.43)	1.56 (0.121)
Depression - Proportion reporting moderate-to-severe levels (score ≥ 10, %)	145	17.9%	145	18.6%	0.05 (0.819)
Anxiety GAD-7 score (0–21)	152	5.14 (5.07)	152	4.15 (4.80)	2.75 (0.007)
Anxiety - Proportion reporting moderate-to-severe levels (score ≥ 10, %)	152	16.4%	152	11.2%	2.91 (0.088)
Mental wellbeing SWEMWBS score (7–35)	153	23.07 (4.37)	153	23.99 (4.94)	−2.72 (0.007)
Mental wellbeing – Proportion reporting a high level of wellbeing (score ≥ 28, %)	153	19.0%	153	25.5%	2.63 (0.105)
Self-rated health (%)	160		160		2.64 (0.451)
*Poor / Fair*		13.1%		14.4%	
*Good*		25.6%		26.3%	
*Very good / Excellent*		61.3%		58.8%	

^a^Paired t-tests for continuous data and McNemar Chi-square tests for proportion data

### Physical activity

There was little change, and no significant differences, between baseline and twelve-month follow-up for importance of being physically active, confidence in being physically active, minutes per week of moderate-to-vigorous-intensity physical activity, or proportion meeting recommended physical activity guidelines (all *p* > 0.05; Table 2). Therefore, linear regression analyses to explore the factors associated with change in physical activity were not conducted.

### Mental health

Neither the continuous PHQ-8 score (*p* = 0.121) nor the proportion of participants reporting moderate-to-severe levels of depression (*p* = 0.819; Table 2) differed significantly between baseline and twelve-month follow-up. Therefore, no further analyses were conducted with depression as the outcome variable. When measured continuously, mean anxiety score reduced from baseline (Mean = 5.14, SD = 5.07) to twelve-month follow-up (Mean = 4.15, SD = 4.80; T = 2.75, *p* = 0.007), but there was no difference in the proportion of participants reporting moderate-to-severe levels of anxiety (*p* = 0.088; Table 2). Mental wellbeing score increased from baseline (Mean = 23.07, SD = 4.37) to twelve-month follow-up (Mean = 23.99, SD = 4.94; T = − 2.72, *p* = 0.007; Table 2). However, there was no difference in the proportion of participants reporting high levels of mental wellbeing (*p* = 0.105).

### Prospective associations of baseline variables with change in healthy eating habit and mental health

#### Diet

In the univariate models, very good/excellent compared to poor/fair self-rated health at baseline was associated with an increase in healthy eating habit strength at 12-month follow-up (ß = 1.27, 95% CI = 0.27 to 2.28). People’s perceived importance (ß = − 0.20, 95% CI = − 0.35 to − 0.05) and confidence (ß = − 0.15, 95% CI = − 0.27 to − 0.04) of eating healthily during the first COVID-19 lockdown at baseline were both associated with a decrease in their healthy eating habit strength at 12-month follow-up.

In the multivariate models, good (ß = 1.23, 95% CI = 0.20 to 2.25) and very good/excellent (ß = 1.71, 95% CI = 0.72 to 2.69) vs poor/fair self-rated health at baseline was associated with an increase in healthy eating habit strength at 12-month follow-up. I.e., the more people rated their health highly during the first lockdown, the stronger their healthy eating habits were after 12-months. People’s perceived importance (ß = − 0.15, 95% CI = − 0.30 to − 0.01, *p* = 0.072) and confidence (ß = − 0.15, 95% CI = − 0.29 to − 0.02, *p* = 0.028) of eating healthily during the first COVID-19 lockdown at baseline were both associated with a reduction in their healthy eating habits at 12-months follow-up (Table 3). I.e., the more confident and important people felt healthy eating was during the first lockdown, the weaker their healthy eating habits were after 12-months. This association with a reduction in healthy eating habit could indicate a ceiling effect, given that both importance and confidence were relatively high at baseline.

**Table 3:** Prospective associations of demographic and diet variables at baseline with change in healthy eating habit at 12-month follow-up

	Univariate Models		Multivariate Model	
	Coefficient (95% CI)	***p***	Coefficient (95% CI)	***p***
Gender		0.508		
*Female*	Reference			
*Male*	− 0.18 (− 0.73 to 0.36)			
Age category		0.647		
*18–34 years*	Reference			
*35–64 years*	− 0.13 (− 0.70 to 0.44)			
*65+ years*	0.22 (−0.63 to 1.06)			
Index of Multiple Deprivation quintile		0.077		
*1–2 (less deprived)*	Reference			
*3, 4 & 5 (more deprived)*	0.48 (−0.05 to 1.00)			
Self-rated health during first COVID-19 lockdown		0.033		0.002
*Poor / Fair*	Reference		Reference	
*Good*	0.94 (−0.12 to 2.00)		1.23 (0.20 to 2.25)	
*Very good / Excellent*	1.27 (0.27 to 2.28)		1.71 (0.72 to 2.70)	
Classed as high risk for COVID-19		0.578		
*No*	Reference			
*Yes*	−0.18 (−0.81 to 0.45)			
Living situation during first COVID-19 lockdown		0.758		
*Living alone*	Reference			
*Living with others (not dependents)*	−0.25 (−0.96 to 0.47)			
*Living with children aged 12–17*	−0.63 (−1.80 to 0.54)			
*Living with children aged 0–11*	−0.25 (− 1.12 to 0.62)			
*Living with someone at risk of COVID-19*	0.06 (−0.82 to 0.94)			
Working situation during first COVID-19 lockdown		0.821		
*Not working*	Reference			
*Working from home*	0.07 (−0.51 to 0.64)			
*Working outside home, but socially distanced*	−0.33 (−1.17 to 0.51)			
*Frontline NHS workers or key workers*	−0.08 (− 0.92 to 0.76)			
Importance of eating healthily during first COVID-19 lockdown	−0.20 (− 0.35 to − 0.05)	0.011	−0.15 (− 0.30 to 0.01)	0.072
Confidence in eating healthily during first COVID-19 lockdown	−0.15 (− 0.27 to − 0.04)	0.007	−0.15 (− 0.29 to − 0.02)	0.028
Perceived change in diet during first COVID-19 lockdown		0.078		0.451
*Diet worsened*	0.69 (0.09 to 1.30)		0.30 (−0.35 to 0.96)	
*Diet stayed the same*	Reference		Reference	
*Diet improved*	0.09 (−0.51 to 0.69)		0.29 (−0.29 to 0.87)	

Sample sizes for the univariate models ranged from 116 to 132; sample size for the multivariate model was 131

#### Anxiety

In the univariate models, perceiving your mental health had worsened during the first lockdown compared to staying the same was associated with a reduction in symptoms of anxiety at 12-month follow-up (ß = − 3.25, 95% CI = − 4.72 to − 1.78). Living with children aged between 12 and 17 vs living alone during the first lockdown was associated with an increase in symptoms of anxiety at 12-months follow-up (ß = − 4.50, 95% CI = 1.13 to 7.88) (model approaching significance at *p* = 0.060).

In the multivariate models, perceiving your mental health had worsened during the first lockdown compared to staying the same was still associated with a reduction in symptoms of anxiety at 12-month follow-up (ß = − 3.05, 95% CI = − 4.53 to − 1.57). Living with children aged between 12 and 17 vs living alone during the first lockdown was also still associated with an increase in symptoms of anxiety at 12-months follow-up (ß = − 3.99, 95% CI = 0.77 to 7.21). However, the overall model was not significant (Table 4).

**Table 4:** Prospective associations of demographic and mental health variables at baseline with change in anxiety score

	Univariate Models		Multivariate Model	
	Coefficient (95% CI)	***p***	Coefficient (95% CI)	***p***
Gender		0.180		
*Female*	Reference			
*Male*	1.11 (− 0.52 to 2.74)			
Age category		0.315		
*18–34 years*	Reference			
*35–64 years*	1.30 (−0.45 to 3.06)			
*65+ years*	1.32 (− 0.91 to 3.55)			
Index of Multiple Deprivation quintile		0.135		
*1–2 (less deprived)*	Reference			
*3, 4 & 5 (more deprived)*	−1.22 (−2.83 to 0.39)			
Self-rated health during first COVID-19 lockdown		0.968		
*Poor / Fair*	Reference			
*Good*	−0.06 (−3.06 to 2.94)			
*Very good / Excellent*	−0.24 (− 3.06 to 2.57)			
Classed as high risk for COVID-19		0.751		
*No*	Reference			
*Yes*	−0.28 (−2.02 to 1.46)			
Living situation during first COVID-19 lockdown		0.060		0.109
*Living alone*	Reference		Reference	
*Living with others (not dependents)*	−0.21 (−2.23 to 1.82)		−0.02 (−1.96 to 1.91)	
*Living with children aged 12–17*	4.50 (1.13 to 7.88)		3.99 (0.77 to 7.21)	
*Living with children aged 0–11*	0.37 (−2.14 to 2.87)		0.72 (−1.67 to 3.12)	
*Living with someone at risk of COVID-19*	0.40 (−2.04 to 2.83)		0.61 (−1.70 to 2.93)	
Working situation during first COVID-19 lockdown		0.705		
*Not working*	Reference			
*Working from home*	−0.60 (−2.25 to 1.05)			
*Working outside home, but socially distanced*	0.56 (−1.91 to 3.03)			
*Frontline NHS workers or key workers*	−0.90 (−3.31 to 1.51)			
Perceived change in mental health during first COVID-19 lockdown		< 0.001		< 0.001
*Mental health worsened*	−3.25 (−4.72 to −1.78)		− 3.05 (−4.53 to − 1.57)	
*Mental health stayed the same*	Reference		Reference	
*Mental health increased*	0.09 (−2.38 to 2.56)		0.21 (− 2.25 to 2.67)	

Sample sizes for the univariate models ranged from 133 to 152; sample size for the multivariate model was 151

#### Mental wellbeing

In the univariate models, compared with being 18–34 years old, being 35–64 years was associated with decrease in mental wellbeing between baseline and follow-up (ß = − 1.70, 95% CI = − 3.32 to − 0.08). However, the overall regression model was non-significant (*P* = 0.112). Perceiving your mental health had worsened during the first lockdown compared to staying the same was associated with an improvement in mental wellbeing (ß = 2.55, 95% CI = 1.17 to − 3.94). In the multivariate models perceiving mental health had worsened compared to staying the same was associated with an increase in mental wellbeing score at 12-months (ß = 2.35, 95% CI = 0.94 to 3.77) (Table 5). I.e., people who felt their mental health had worsened during lockdown had improved mental wellbeing a year later.

**Table 5:** Prospective associations of demographic and mental health variables at baseline with change in mental wellbeing

	Univariate Models		Multivariate Model	
	Coefficient (95% CI)	***p***	Coefficient (95% CI)	***p***
Gender		0.094		
*Female*	Reference			
*Male*	−1.29 (− 2.81 to 0.22)			
Age category		0.112		0.308
*18–34 years*	Reference		Reference	
*35–64 years*	−1.70 (−3.32 to −0.08)		−1.19 (−2.78 to 0.41)	
*65+ years*	− 0.89 (−2.91 to 1.13)		− 0.49 (− 2.47 to 1.49)	
Index of Multiple Deprivation quintile		0.127		
*1–2 (less deprived)*	Reference			
*3, 4 & 5 (more deprived)*	1.12 (−0.32 to 2.56)			
Self-rated health during first COVID-19 lockdown		0.321		
*Poor / Fair*	Reference			
*Good*	−1.16 (−3.93 to 1.61)			
*Very good / Excellent*	−0.04 (−2.65 to 2.58)			
Classed as high risk for COVID-19		0.782		
*No*	Reference			
*Yes*	−0.22 (−1.81 to 1.37)			
Living situation during first COVID-19 lockdown		0.534		
*Living alone*	Reference			
*Living with others (not dependents)*	0.13 (−1.76 to 2.02)			
*Living with children aged 12–17*	−2.48 (−5.67 to 0.70)			
*Living with children aged 0–11*	−0.26 (− 2.64 to 2.11)			
*Living with someone at risk of COVID-19*	−0.04 (−2.27 to 2.19)			
Working situation during first COVID-19 lockdown		0.846		
*Not working*	Reference			
*Working from home*	0.08 (−1.45 to 1.61)			
*Working outside home, but socially distanced*	0.62 (−1.76 to 3.00)			
*Frontline NHS workers or key workers*	−0.72 (−3.04 to 1.60)			
Perceived change in mental health during first COVID-19 lockdown		< 0.001		0.002
*Mental health worsened*	2.55 (1.17 to 3.94)		2.35 (0.94 to 3.77)	
*Mental health stayed the same*	Reference		Reference	
*Mental health increased*	−0.83 (−3.17 to 1.51)		−0.90 (− 3.26 to 1.45)	

Sample sizes for the univariate models ranged from 135 to 153; sample size for the multivariate model was 153

## Discussion

Concerning the first research question, the present study found that during the initial lockdown, participants were generally active and had good eating habits. However, as least one out of five reported moderate to severe levels of depression and anxiety. For the second research question, over the 12 months, we found that healthy eating habit strength, and the importance of and confidence in eating healthily, were all reduced. Conversely, anxiety scores reduced and well-being increased. For the third research question, we found that self-rated health and confidence in eating healthily at baseline were positively and negatively associated with a 12-month change in healthy eating habits, respectively. Living with children aged 12–17 (compared to living alone) was associated with an increase in anxiety while perceiving mental health to have worsened during the first lockdown (compared to staying the same) was associated with reduced anxiety. Perceiving mental health to have worsened initially (compared to staying the same) was associated with an increase in mental wellbeing.

In this study, we found that in the 12 months since the start of the UK COVID-19 lockdown restrictions, the psycho-social variables related to healthy eating (habit, importance, and confidence) worsened across time. This is a concern, especially considering that a greater proportion of participants perceived their diet had worsened (compared to improved) at the start of the first lockdown restrictions (23.1% versus 19.0%). The associations between the change in the strength of healthy eating habits and participants’ self-rated health at baseline, suggested that this negative impact may be more prevalent for participants in fair or poor physical health at the outset. It is possible that some participants felt more confident in their ability to eat healthily when lockdown restrictions were tighter, when they had more time and opportunity for cooking healthy meals, and there were fewer opportunities to eat out in social settings. Our data also indicated that living with secondary school aged children experienced worsening anxiety relative to people with younger or no children who showed no change.

Early research during the COVID-19 pandemic suggested an increase in consumption of unhealthy food, eating out of control and snacking between meals increased during the initial COVID-19 lockdown measures [14]. However, in contrast to this, we found healthy eating habit, importance and confidence of eating healthy dropped between lockdown and 12-months later. This is also supported by a short-term longitudinal study of Italian adults (*N* = 728) examining eating styles and behaviours between April 2020 (during lockdown) and June 2020 (after lockdown). The researchers found that during lockdown, participants reported an increase in healthy food consumption, involvement in cooking, and a decrease in junk food consumption [33]. Whereas, in the post-lockdown period, participants cut down their healthy food consumption and their involvement in food preparation but continued to reduce their junk food intake [33]. Time constraints and lack of willpower are well-known barriers to healthy eating [34], therefore, removing these barriers may result in healthier eating habits. However, when these barriers were restored as lockdown restrictions were eased, the opportunity for unhealthy habits to return increased. Our finding that higher perceived importance and confidence in healthy eating was associated with weaker habit at 12 months was unexpected as this contrasts with usual directions of effect; further work to explore hypotheses for this pattern is warranted.

Among our sample, there was variation in the degree to which participants believed their physical activity behaviour to have changed at the onset of COVID-19 lockdown restrictions, with approximately one third of participants perceiving their physical activity to have increased, stayed the same, or decreased respectively. This somewhat contradicts some of the earlier published studies that observed reductions in physical activity through the start of the pandemic [14–16]. While many recreational and sports facilities were closed at the onset of the pandemic which limited activity choice and opportunities, government messaging highlighted exercise as one of the only reasons permissible for leaving the house, which may have increased motivation to be active for some individuals [35].

Similar to our findings, an international cross-sectional survey study (*N* = 13,696) conducted in March–May 2020, found that 44.2% of participants reported no change, 23.7% reported a decrease, and 31.9% reported an increase in their exercise frequency during the COVID-19 pandemic [36]. The authors also developed a prediction model to estimate changes in exercise frequency in future lockdowns, with results suggesting that those who rarely exercise before a lockdown tend to increase their exercise frequency during it, while those who are frequent exercisers before a lockdown tend to maintain it [36]. This variation in behaviour may explain why we found no difference in physical activity between baseline (during the first lockdown) and twelve-months later (when restrictions had started to be eased); we did not have a pre-lockdown measure of physical activity to enable us to test this interaction. Future longitudinal research would be useful to explore how the pandemic and the subsequent lockdown restrictions have had differential effects on the physical activity behaviour of specific population sub-groups to ensure interventions can be appropriately targeted.

The impact of COVID-19 lockdown restrictions on mental health has been a major concern [37], with research suggesting that levels of mental distress, anxiety, and depression increased at the onset of the pandemic [19–21,[33]]. A cross-sectional study of UK adults (*N* = 3097) measuring mental health at the start of the pandemic (April 2020), found 31.6% reported moderate-to-severe levels of depression and 26% reported moderate-to-severe levels of anxiety [38]. While levels of depression and anxiety were slightly lower in the present study (25.3 and 20.5% respectively), both studies indicate mean levels of depression and anxiety during the start of the COVID-19 pandemic exceed previously published population norms [39, 40]. Our study expands this data, but demonstrating that levels of depression appeared to be consistent within our sample, but anxiety and mental wellbeing appeared to improve across time, suggesting that any negative effects of the pandemic on mental health may be reversible. Indeed, compared to those who perceived their mental health had stayed the same, participants who perceived their mental health had initially worsened during the first COVID-19 lockdown were more likely to report improvements in anxiety and mental wellbeing at the twelve-month follow-up. This ‘bounce-back’ effect for anxiety and mental wellbeing may have been due to the easing of restrictions, which enabled increased freedom to see family and friends, participate in hobbies and allow some individuals to return to work (lessening financial insecurity). There is a well-established link between physical activity and mental health which has remained during the COVID-19 pandemic [41]. However, the present study suggests that, given the lack of change in physical activity over 12 months, improvements in anxiety and mental well-being were not driven by physical activity. A previous study in Canada showed that walking and exercise were cited among the top four activities that people engaged in during the COVID-19 pandemic meaning that people continued to find ways to be active, despite restricted opportunities [42].

However, our findings do suggest that anxiety deteriorated for people living with 11–17 year older children. Reasons for this warrant further investigation, including whether this reflects parents’ concerns about the continued disruption of education that persisted throughout the 12 months following the first lockdown, their anxiety at having to manage home-education while fulfilling their own work commitments, or other factors such as concern on the long term impact of the ongoing restrictions on their children’s health and wellbeing.

Similar findings have been shown internationally. A large population-based survey study in China (*N* = 105,248) found that the prevalence of being high risk for mental disorders decreased from 25.8% when lockdown restrictions were in place (early-February 2020) to 20.9% when most COVID-19 restrictions were eased (mid-March 2020) [43]. However, it is still unknown whether this ‘bounce-back’ effect is present across all population sub-groups, or whether the mental health of certain groups remain negatively impacted by the COVID-19 pandemic.

## Strengths and limitations

Strengths of this study include the longitudinal design, with data collected during the first UK COVID-19 lockdown restrictions and twelve-months later at the same time of year, overcoming the issue of the seasonal variation in physical activity, food intake, and mental health [44–47]. This study measured multiple domains of lifestyle behaviours and mental health using validated measures [23, 27, 28], as well as relevant psychosocial factors and demographic variables, allowing us to explore which groups were most susceptible to change in lifestyle behaviours.

The study sample was relatively homogenous, primarily female, of White British origin from South-West England, which limits the ability to extrapolate to other ethnic groups in more diverse areas of the UK. Only one quarter of the baseline sample completed the twelve-month follow-up survey. We were not able to analyse diet behaviour. However, healthy eating habit was included as a proxy for diet behaviour because it has previously been found to be strongly correlated with dietary behaviour [26]. While the IPAQ-SF has been found to have acceptable levels of validity and reliability [23], it typically overestimates physical activity behaviour [48], which may explain the high levels of physical activity among our sample. A further limitation of this study is that sleep was not assessed. Sleep quality has been positively associated with better mental health [49–51]. Furthermore, physical activity has been shown to benefit sleep quality and quantity [52]. Our sample showed higher than average levels of physical activity. However, we were unable to test if their activity levels were associated with sleep and, in turn, mental health. We were also not able to capture lifestyle behaviours and mental health prior to the onset of the pandemic, thus we were reliant on participants’ perceptions of how these variables changed at the onset of the COVID-19 lockdown measures, which provided us with an indication of direction but not the magnitude of change. Finally, this study only took a snapshot of two points in time which does not fully reflect the fluctuating nature of pandemic lockdowns over time.

Further follow-up and monitoring of diet, physical activity and mental health is needed to understand the long-term impact of the COVID-19 lockdown restrictions both in the UK and worldwide. More longitudinal studies are needed to investigate the factors associated with change in lifestyle behaviours and mental health, to highlight whether there are any specific population sub-groups who have been particularly negatively impacted by the COVID-19 lockdown restrictions. Such data will help to identify relevant interventions and/or government policies that could be developed and implemented to combat any negative impacts of the COVID-19 pandemic and ensure that any positive impacts are capitalised on. Finally, more qualitative studies are needed to provide further insight into some of the key drivers of health behaviours both during and after lockdown.

## Conclusions

To our knowledge, this is one of the first studies to report twelve-month follow-up data on the longitudinal impact of the UK COVID-19 lockdown measures on lifestyle behaviours and mental health. We provide evidence that healthy eating habits worsened in the 12 months since the pandemic started, while anxiety and mental wellbeing improved. Participants were more confident in their ability to eat healthily when lockdown restrictions were tighter, potentially due to increased opportunities for home cooking and fewer opportunities to eat out. Participants who perceived their mental health had worsened at the start of the lockdown restrictions were more likely to report positive changes in their level of anxiety and mental wellbeing twelve-months later, suggesting there may be a ‘bounce-back’ effect as restrictions were eased. More longitudinal research is needed into how lifestyle behaviours and mental health have changed since the start of the pandemic, and the factors associated with change, so that effective interventions and government policies can be developed and deployed.

## Supplementary Information

**Additional file 1.**

## Data Availability

The datasets used and/or analysed during the current study are available from the corresponding author on reasonable request.

## Abbreviations

CI: Confidence Interval
COVID-19: Coronavirus-19
GAD-7: General Anxiety Disorder-7 scale
ID: Identifier
IMD: Indices of Multiple Deprivation
IPAQ-SF: International Physical Activity Questionnaire - Short Form
KG: Kilograms
NHS: National Health Service
PHQ-8: Patient Health Questionnaire-8 scale
SD: Standard Deviation
SRBAI: Self-Report Behavioural Automaticity Index
SWEMWBS: Short Warwick-Edinburgh Mental Wellbeing Scale
UK: United Kingdom
UKHLS: UK Household Longitudinal Study
