# Supplementary material for: Life in lockdown: a longitudinal study investigating the impact of the UK COVID-19 lockdown measures on lifestyle behaviours and mental health
Source: BMC Public Health. 2022 Aug 5;22:1495. doi: 10.1186/s12889-022-13888-1 (PMC9354438; doi:10.1186/s12889-022-13888-1)
Supplement: Supplementary file 1 — Additional file 1. [file 12889_2022_13888_MOESM1_ESM.docx]

Survey

**Section 1: Demographics**

1. In which age group do you belong?

18 - 24

25 - 34

35 - 44

45 – 54

55 – 64

1. – 74

75+

1. What is your gender?

Female

Male

Non-binary

Prefer not to say

1. What is your post code? ______________
2. In general, before the COVID-19 outbreak, would you say your health was: (please select one option only)

Excellent

Very good

Good

Fair

Poor

1. And during the period of the UK ‘lockdown’ measures (social distancing and social isolation) would you say your health has been: (please select one option only)

Excellent

Very good

Good

Fair

Poor

1. Have you had COVID-19?

Yes, I developed symptoms and tested positive

I think so, I developed symptoms but was not tested

No

1. Have you been advised that you are (or you consider yourself to be) classed as being ‘at high risk’ in relation to Covid-19

Yes – I am aged 70 years of over

Yes – I have a health condition which classes me as high risk

No

1. If yes – are you self-isolating?

Yes

No

1. Do you have any long-term illness, health problem or disability which limits your daily activities or the work you can do? This includes problems that are due to old age. (please tick one box only)

Yes

No

1. Apart from yourself, who else do you live with (please state how many people in your household within each age category):

For example, if you live with two children aged 6 and 10 you would write ‘2’ next to the 5-11 years category.

0-4 years

5-11 years

12-17 years

18-24 years

25-34 years

35-44 years

45-54 years

65-74 years

75+ years

10. Is anyone in your household (tick all that apply):

on the shielded patient list?

over 70 or living with a chronic health condition that puts them at moderate risk of complications from covid-19?

11. Please provide your employment status prior to the Covid-19 lockdown measures, and any changes to your employment during the Covid-19 lockdown measures:

- 1. *What was your employment status prior to Covid-19 lockdown measures (23^rd^ March 2020)*;

Full time

Part time

Self-employed

Retired

Student

Unemployed

- 1. Prior to Covid-19 did you:
     1. Mainly work from home
     2. Mainly work outside of home (e.g. in an office)
     3. Both work from home and outside of home
  2. *What has changed to your employment status during Covid-19 lockdown measures (please tick any that apply);*

No change

I’ve been furloughed

I have been working from home

I have been made redundant due to Covid-19

My job is precarious due to Covid-19

I’ve had to take a pay cut as a result of Covid-19

Other change (please specify) _________________

1. If you have been working outside your home during Covid-19 lockdown measures, please tell us about the work that you have been doing:

N/A

Frontline worker in the NHS

Key worker unable to maintain social distancing (e.g., supermarket assistant, teacher)

Any other work outside the home, but where you are able to maintain social distancing

1. What is your ethnic group?

*(Choose one option that best describes your ethnic group or background)*

*White*

English/Welsh/Scottish/Northern Irish/British
Irish
Gypsy or Irish Traveller
Any other White background, please describe

*Mixed/Multiple ethnic groups*

White and Black Caribbean
White and Black African
White and Asian
Any other Mixed/Multiple ethnic background, please describe

*Asian/Asian British*

Indian
Pakistani
Bangladeshi
Chinese
Any other Asian background, please describe

*Black/ African/Caribbean/Black British*

African
Caribbean
Any other Black/African/Caribbean background, please describe

*Other ethnic group*

Arab

Any other ethnic group, please describe _________________

**Section 2: Diet**

1. Has your diet changed since lockdown?

No

Yes

Don’t know

IF YES: Has your diet:

Improved during lockdown

Worsened during lockdown

Neither improved nor worsened, just different

IF IMPROVED DURING LOCKDOWN: Do you intend to carry on with the changes to your diet after lockdown?

| Definitely do not | |  |  |  | Definitely do | |
| --- | --- | --- | --- | --- | --- | --- |
| 1 | 2 | 3 | 4 | 5 | 6 | 7 |

IF IMPROVED DURING LOCKDOWN: Why do you feel that your diet has improved since lockdown? (please tick any that apply)

It is harder to access unhealthy foods

I have more time to prepare meals

I have made a deliberate effort to improve my diet

Other (please describe): ________________________

IF WORSENED DURING LOCKDOWN: Do you intend to for your diet to go back to normal after lockdown?

| Definitely do not | |  |  |  | Definitely do | |
| --- | --- | --- | --- | --- | --- | --- |
| 1 | 2 | 3 | 4 | 5 | 6 | 7 |

IF WORSENED DURING LOCKDOWN: Why do you feel that your diet has worsened since lockdown? (please tick any that apply)

Less healthy foods are easy to access

Harder or more expensive to buy healthier foods due to supermarket shortages

I am eating less healthily due to worry (‘comfort eating’)

I have less time to prepare meals

Other (please describe): _________________________

1. Do you have concerns about getting access during lockdown to the food you and your family normally eat?

Yes

No

Don’t know

1. If ‘yes’, please tell us what these concerns are:

_____________________________________

***NEW PAGE***

1. Deciding to eat healthy foods is something…

|  | Completely disagree | | | | Completely agree | | |
| --- | --- | --- | --- | --- | --- | --- | --- |
| I do automatically | 1 | 2 | 3 | 4 | 5 | 6 | 7 |
| I do without having to consciously remember | 1 | 2 | 3 | 4 | 5 | 6 | 7 |
| I do without thinking | 1 | 2 | 3 | 4 | 5 | 6 | 7 |
| I start doing before I realise I’m doing it. | 1 | 2 | 3 | 4 | 5 | 6 | 7 |

1. Eating a healthy diet is something that...

|  | Definitely disagree | | |  | Definitely agree | | |
| --- | --- | --- | --- | --- | --- | --- | --- |
| fits the way I want to live | 1 | 2 | 3 | 4 | 5 | 6 | 7 |
| fits who I am | 1 | 2 | 3 | 4 | 5 | 6 | 7 |
| is how I perceive myself | 1 | 2 | 3 | 4 | 5 | 6 | 7 |
| is typical for me | 1 | 2 | 3 | 4 | 5 | 6 | 7 |

1. How important do you think it is to eat a healthy diet?

| not at all important | |  |  |  |  |  |  | very  important | |
| --- | --- | --- | --- | --- | --- | --- | --- | --- | --- |
| 1 | 2 | 3 | 4 | 5 | 6 | 7 | 8 | 9 | 10 |

1. How important do you think it is to eat a healthy diet during the lockdown period?

| not at all important | |  |  |  |  |  |  | very  important | |
| --- | --- | --- | --- | --- | --- | --- | --- | --- | --- |
| 1 | 2 | 3 | 4 | 5 | 6 | 7 | 8 | 9 | 10 |

1. How confident are you that you can eat a healthy diet during this time in lockdown?

| not at all confident | |  |  |  |  |  |  | very  confident | |
| --- | --- | --- | --- | --- | --- | --- | --- | --- | --- |
| 1 | 2 | 3 | 4 | 5 | 6 | 7 | 8 | 9 | 10 |

**Section 3: Physical Activity**

1a. During the last 7 days, on how many days did you walk for at least 10 minutes at a time?

This includes walking at home, walking to travel from place to place, and any other walking that you did solely for recreation, exercise or leisure. (Do not include walking you did at work)

Days (per week) _________ *Write in, put ‘0’ if none*

1b. How much time in total did you usually spend walking on one of those days?

*Write in _______ hours _______ minutes*

2. How would you describe your usual walking pace?

*(please select one answer)*

Slow

Steady

Brisk

Fast

3a. During the last 7 days, on how many days did you do moderate physical activities? Examples of moderate activities include carrying light loads and bicycling at a regular pace.

Again, think only about those physical activities that you did for at least 10 minutes at a time. (Do not include walking or work activities)

Days (per week) _________ *Write in, put ‘0’ if none*

3b. How much time in total did you usually spend on one of those days doing moderate physical activities?

*Write in _______ hours _______ minutes*

4a. During the last 7 days, on how many days did you do vigorous physical activities? Examples of these activities include heavy lifting, digging, aerobics, running and fast bicycling.

Think about only those physical activities that you did for at least 10 minutes at a time, and do not include work activities.

Days (per week) _________ *Write in, put ‘0’ if none*

4b. How much time in total did you usually spend on one of those days doing vigorous physical activities?

*Write in _______ hours _______ minutes*

5. This question is about the time you spent sitting on weekdays while travelling, at home and during leisure time.

This includes time spent sitting at a computer, travelling in a car, or sitting or lying down to watch television.

During the last 7 days, how much time in total did you usually spend sitting on a week day? (Do not include time spent sitting at work)

*Write in _______ hours _______ minutes*

6. Please tell us the type and amount of activity involved in your work. Only include activity involved in work you are currently doing (I.e., in the last 7 days)

(please select one answer only)

I am not in employment (e.g., retired, unemployed, studying, furloughed etc.)

I spend most of my time at work sitting (such as working from home or in an office)

I spend most of my time at work standing or walking. However, my work does not require much intense physical effort

My work involves definite physical effort including handling of heavy objects and use of tools

7. Has your physical activity/exercise changed since lockdown?

No

Yes

IF YES: Since lockdown, has your physical activity/exercise:

Increased

Decreased

Neither increased nor decreased, just different

IF INCREASED: Why do you think that your physical activity/exercise has increased since lockdown?

Change in working conditions to sit less

Additional/new responsibilities keeping me active (e.g., childcare, volunteering)

Helps prevent boredom

More time available to exercise

Other (please specify) _______________

IF INCREASED: Do you intend to carry on with the changes after lockdown?

| Definitely do not | |  |  |  | Definitely do | |
| --- | --- | --- | --- | --- | --- | --- |
| 1 | 2 | 3 | 4 | 5 | 6 | 7 |

IF INCREASED: What do you think would help you to achieve this? ___________________

IF DECREASED: Why do you think that your physical activity/exercise decreased during lockdown?

Unable to access usual facilities/open spaces

Worry about social distancing

Illness/injury limits movement

Lack of motivation

Lack of time

Other: _______________________

IF DECREASED: Do you intend for your physical activity to go back to normal after lockdown?

| Definitely do not | |  |  |  | Definitely do | |
| --- | --- | --- | --- | --- | --- | --- |
| 1 | 2 | 3 | 4 | 5 | 6 | 7 |

IF DECREASED: What do you think would help you to achieve this?

___________________________________________

8.Have you sought any physical activity/exercise resources during lockdown for your personal use (e.g. exercise videos on YouTube)?

No

Yes

8a. If ‘yes’, What resources have you used?

8b. If ‘yes’, are you regularly using these resources (e.g., at least once per week)?

No

Yes

8c. If ‘yes’, Do you think you will continue with this activity after lockdown?

Yes

No

Maybe

1. How confident are you that you can be physically active enough for your health over the lockdown period?

| not at all confident | |  |  |  |  |  |  | very  confident | |
| --- | --- | --- | --- | --- | --- | --- | --- | --- | --- |
| 1 | 2 | 3 | 4 | 5 | 6 | 7 | 8 | 9 | 10 |

1. How important do you think it is to be physically active over the lockdown period?

| not at all important | |  |  |  |  |  |  | very  important | |
| --- | --- | --- | --- | --- | --- | --- | --- | --- | --- |
| 1 | 2 | 3 | 4 | 5 | 6 | 7 | 8 | 9 | 10 |

1. Deciding to take daily physical activity/exercise is something…

|  | Completely disagree | | | | Completely agree | | |
| --- | --- | --- | --- | --- | --- | --- | --- |
| I do automatically | 1 | 2 | 3 | 4 | 5 | 6 | 7 |
| I do without having to consciously remember | 1 | 2 | 3 | 4 | 5 | 6 | 7 |
| I do without thinking | 1 | 2 | 3 | 4 | 5 | 6 | 7 |
| I start doing before I realise I’m doing it. | 1 | 2 | 3 | 4 | 5 | 6 | 7 |

1. Engaging in physical activity/exercise is something that...

|  | Definitely disagree | | |  | Definitely agree | | |
| --- | --- | --- | --- | --- | --- | --- | --- |
| fits the way I want to live | 1 | 2 | 3 | 4 | 5 | 6 | 7 |
| fits who I am | 1 | 2 | 3 | 4 | 5 | 6 | 7 |
| is how I perceive myself | 1 | 2 | 3 | 4 | 5 | 6 | 7 |
| is typical for me | 1 | 2 | 3 | 4 | 5 | 6 | 7 |

**Section 4: Mental Health**

| **Over the last 2 weeks, how often have you been bothered by any of the following problems?** | **Not at all** | **Several days** | **More than half the days** | **Nearly every day** |
| --- | --- | --- | --- | --- |
| Little interest or pleasure in doing things | 0 | 1 | 2 | 3 |
| Feeling down, depressed, or hopeless | 0 | 1 | 2 | 3 |
| Trouble falling or staying asleep, or sleeping too much | 0 | 1 | 2 | 3 |
| Feeling tired or having little energy | 0 | 1 | 2 | 3 |
| Poor appetite or overeating | 0 | 1 | 2 | 3 |
| Feeling bad about yourself – or that you are a failure or have let yourself or your family down | 0 | 1 | 2 | 3 |
| Trouble concentrating on things, such as reading the newspaper or watching television | 0 | 1 | 2 | 3 |
| Moving or speaking so slowly that other people could have noticed? Or the opposite – being so fidgety or restless that you have been moving around a lot more than usual | 0 | 1 | 2 | 3 |

| **Over the last 2 weeks, how often have you been bothered by any of the following problems?** | **Not at all** | **Several days** | **More than half the days** | **Nearly every day** |
| --- | --- | --- | --- | --- |
| Feeling nervous, anxious or on edge | 0 | 1 | 2 | 3 |
| Not being able to stop or control worrying | 0 | 1 | 2 | 3 |
| Worrying too much about different things | 0 | 1 | 2 | 3 |
| Trouble relaxing | 0 | 1 | 2 | 3 |
| Being so restless that it is hard to sit still | 0 | 1 | 2 | 3 |
| Becoming easily annoyed or irritable | 0 | 1 | 2 | 3 |
| Feeling afraid as if something awful might happen | 0 | 1 | 2 | 3 |

1. Below are some statements about feelings and thoughts. Please tick the box that best described your experience of each over the last 2 weeks.

|  | **None of the time** | **Rarely** | **Some of the time** | **Often** | **All of the time** |
| --- | --- | --- | --- | --- | --- |
| I’ve been feeling optimistic about the future | 1 | 2 | 3 | 4 | 5 |
| I’ve been feeling useful | 1 | 2 | 3 | 4 | 5 |
| I’ve been feeling relaxed | 1 | 2 | 3 | 4 | 5 |
| I’ve been dealing with problems well | 1 | 2 | 3 | 4 | 5 |
| I’ve been thinking clearly | 1 | 2 | 3 | 4 | 5 |
| I’ve been feeling close to other people | 1 | 2 | 3 | 4 | 5 |
| I’ve been able to make up my own mind about things | 1 | 2 | 3 | 4 | 5 |

1. **Has your mental health changed since lockdown?**

Yes

No

Don’t know

IF YES: Has your mental health:

Improved

Worsened

Neither improved nor worsened, just different

Please explain why?

1. **Are you doing anything in particular to try and help your mental health?**

Yes

No

Don’t know

1. If ‘yes’, please tell us what you have been doing:

_____________________________________

Thank you for completing this survey. If you have any questions, please do not hesitate to contact the research team on the contact details below.

If you require support for your mental health this can be accessed here:

<https://www.samaritans.org/>

<https://www.rethink.org/aboutus/what-we-do/advice-and-information-service/get-help-now/>
